# Full genome sequencing of archived wild type and vaccine rinderpest virus isolates prior to their destruction

**DOI:** 10.1038/s41598-020-63707-z

**Published:** 2020-04-16

**Authors:** Simon King, Paulina Rajko-Nenow, Honorata M. Ropiak, Paolo Ribeca, Carrie Batten, Michael D. Baron

**Affiliations:** 1 The Pirbright Institute, Ash Road, Pirbright, Surrey, GU24 0NF UK; 2 Present Address: Biomathematics and Statistics Scotland, JCMB, The King’s Buildings, Peter Guthrie Tait Road, Edinburgh, EH9 3FD Scotland UK

**Keywords:** Viral evolution, Pathogens, Vaccines

## Abstract

When rinderpest virus (RPV) was declared eradicated in 2011, the only remaining samples of this once much-feared livestock virus were those held in various laboratories. In order to allow the destruction of our institute’s stocks of RPV while maintaining the ability to recover the various viruses if ever required, we have determined the full genome sequence of all our distinct samples of RPV, including 51 wild type viruses and examples of three different types of vaccine strain. Examination of the sequences of these virus isolates has shown that the African isolates form a single disparate clade, rather than two separate clades, which is more in accord with the known history of the virus in Africa. We have also identified two groups of goat-passaged viruses which have acquired an extra 6 bases in the long untranslated region between the M and F protein coding sequences, and shown that, for more than half the genomes sequenced, translation of the F protein requires translational frameshift or non-standard translation initiation. Curiously, the clade containing the lapinised vaccine viruses that were developed originally in Korea appears to be more similar to the known African viruses than to any other Asian viruses.

## Introduction

Rinderpest (RP) was one of the most severe diseases of cattle ever recorded, with high morbidity rates, and mortality rates of 80% to 90% in naïve populations. The disease was declared eradicated in 2011^1^, thus becoming the second viral disease, after smallpox, to be eradicated, with global benefits estimated to be in the billions of dollars^2^. The RP virus (RPV) itself has not been entirely eliminated, with a number of laboratories known to have samples of wild type RPV. Accidental release of RPV from such a laboratory is thought to be the most likely pathway by which the virus might re-enter the environment^3,4^, although it might also be deliberately released as an act of sabotage or bioterrorism. The member states of the World Organisation for Animal Health (OIE), and the Food and Agricultural Organisation of the United Nations (FAO) agreed to restrict all work with the virus and to allow the storage of the virus only in highly secure Rinderpest Holding Facilities (RHFs) that have been inspected and approved jointly by OIE and FAO.

The FAO-OIE RHF in the UK is the Pirbright Institute which, as the Institute for Animal Health, and before that the Animal Virus Research Institute, was a centre for research on RPV since the 1960s. The institute developed the monoclonal antibody-based competition ELISA (cELISA) used extensively for surveillance^5^, as well as the most widely used RT-PCR assay for RPV^6^ and the concept of different geographic lineages of the virus based on the sequence of the product from the RT-PCR^7^. The first RPV genome sequences were determined at Pirbright^8,9^ and the system for recovering RPV from a cDNA copy of the genome was developed there^10^. Because of this history, and its links to many of the countries where RPV was still endemic in the latter half of the 20^th^ century, the institute had accumulated a significant number of RPV isolates from a range of countries. Some of these were tissue samples while other isolates had been grown in cell culture for various research purposes. Most of these isolates had not been subjected to extensive characterisation, either as pathogens or at the molecular level. Full genome sequences have only been determined for the “Plowright” tissue culture-attenuated vaccine strain^8^ and the virulent virus from which it was derived^9^. Simply destroying all stocks of wild type virus would pose the risk that information would be lost that might one day be useful, since other uncharacterised morbilliviruses are known to exist^11^ and may have the potential to move into the environmental niche presented by a global population of cattle lacking immunity to these viruses. One way to mitigate this risk would be to sequence the genomes of these viruses prior to their destruction. The system for recovery of live RPV from a copy of its genome^10^ is well-established, and has been used to create a large number of recombinant RPVs over the years e.g.^12–16^. Current DNA synthesis techniques are such that a complete RPV genome could be built into the appropriate plasmid, as was recently done to recover live peste des petits ruminants virus (PPRV) from a cDNA copy of the genome built entirely *de novo*^17^. Determining the full genome sequence of viruses in our existing archive would enable any of those viruses to be recreated should they ever be required in the future, meaning it was no longer necessary to keep that actual virus. It would also provide a database of sequences that might be useful in tracing the origin of any potential future outbreak of RP, as well as information about the evolution of the virus over time. The project to determine these sequences, followed by destruction of the virus samples was therefore proposed to, and approved by, OIE and FAO. We present here the results of that project, which have identified several new and unexpected features of the genome of some RPV isolates, as well as improving our understanding of the spread of the virus in Africa.

## Results and Discussion

### Sequencing libraries

**Figure 1:**
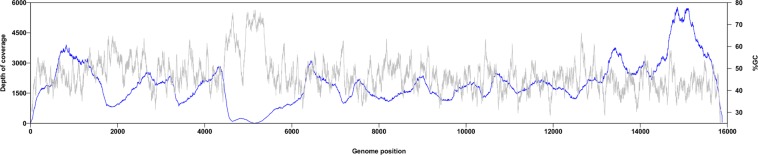
Average depth of sequencing coverage from MiSeq data. The depth of coverage was determined for each genome after mapping against the consensus sequence for that isolate, and the average depth of coverage over all genomes is shown in blue. The % GC was determined for each genome at each position along the genome from 29–15854 using a sliding 59-base window, and the average % GC taken over all genomes is plotted in grey.

All samples were first screened by reverse transcription real time PCR (RT-qPCR) specific for RPV^18^. RPV-positive RNA samples were processed to create sequencing libraries which were sequenced using an Illumina MiSeq. RPV is a morbillivirus, an enveloped RNA virus with a negative sense genome of 15882 bases^8^. As with all the morbilliviruses, it is pleiomorphic and cannot be easily purified free of host cell material^19,20^. Both tissue samples and cultured virus were expected to contain a high percentage of host cell RNA. Libraries prepared using standard Nextera kits from cell-cultured RPV contained a highly variable fraction of RPV RNA (median: 5%; range: 0.01–70%), while for tissue samples, even those giving similarly low Ct in the RT-qPCR assay (indicating high RPV content), the fraction of total RNA derived from RPV was lower (median: 0.16%; range: 0–84%). Because of the uneven distribution of reads along the genome (Fig. 1), at least 3000 read pairs were required to give good coverage of the majority of the genome, and most tissue samples gave many fewer RPV-specific reads than this.

Several techniques were used in attempts to improve the fraction of total tissue RNA that was derived from RPV. The most effective technique was amplification of total RNA using a single primer isothermal amplification system (SPIA), followed by partial depletion of host rRNA using a human sequence-optimised system (see Methods), giving an approximately 35-fold improvement (mean 34.72, s.d. = 18.7) in the specific RPV content in the MiSeq libraries for the samples that were analysed by both methods. Supplementary Table S1 gives the sample preparation method and sequencing results (total number of reads and number matching RPV) for each sample sequenced.

### Assembling RPV genome sequences

RPV genome assembly was performed by mapping the sequence data to an existing RPV genome, the wild type RPV Kabete ‘O’ sequence^9^ (RPV-KO). The program *bowtie2*^21^ was the most effective at identifying reads mapping to heterologous RPV isolates, while *bwa-mem*^22^ was more effective at identifying and incorporating data from reads that were derived from viral copy-back RNAs. Since all reads covering the ends of the genome were derived from such copy-back RNAs, each program provided information not available with the other. Both mappers were therefore used, combining the information to give the final consensus sequence. Assembling the RPV genome by *de novo* assembly using any of several existing programs was not as effective.

Average sequence coverage from the NGS data is shown in Fig. 1. For almost all isolates, one or two regions of the long GC-rich M-F UTR were not determined from the NGS data, even when sequencing the RPV-KO isolate itself, so this was not a problem caused by mapping to a heterologous RPV isolate. Given that the sections of genome not found in the sequencing library were among the most GC-rich sections of the genome sequence (Fig. 1), it is likely that this problem was due to a failure of cDNAs containing these sequences to be effectively amplified during the PCRs used to attach barcodes and adapters during library preparation, as has previously been reported^23^. The genome sequence in these regions was therefore determined by RT-PCR and sequencing the products by Sanger sequencing. Where the 5′ end or the 3′ end of the genome were not recovered from the Miseq dataset, the missing information was obtained by RACE (see Methods) and Sanger sequencing.

### Sequence features in the completed genomes

A total of 121 full genomes were determined plus 2 more genomes lacking only ~40 bases at the 5′ end. Of these 123, 10 were preparations of the RBOK vaccine strain from different sources and a further 11 genomes were preparations of lapinised RPV in use at different RP research laboratories, specifically the East African Veterinary Research Organisation at Muguga, Kenya (EAVRO), the Plum Island Animal Disease Centre in the USA (PIADC) and the predecessor of the Pirbright Institute, the Animal Virus Research Institute (AVRI), while 11 were preparations of goat-adapted vaccine from different sources. The remaining 91 genomes represented 51 discrete isolates of wild type RPV.

**Figure 2:**
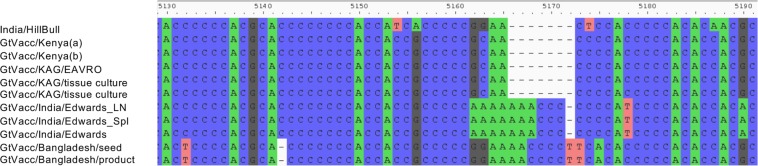
Extra bases found in the genomes of specific goat-adapted RPVs. The sequence at the relevant portion of the F gene is shown for all goat-adapted RPV genomes, plus the oldest sample of RPV from India in our dataset (RPV/India/HillBull).

All the genomes were 15882 bases long, as previously reported^8,9,24–27^, except for 5 samples of goat-adapted vaccine virus (GtVacc), each of which had an extra 6 bases in the long GC-rich 5′ UTR of the F gene (Fig. 2). These samples could be divided into two groups, GtVacc from Bangladesh (vaccine seed and production vaccine) and a sample of GtVacc from India; for the latter, we sequenced some of the original material and also freeze-dried tissue from a UK goat that had been inoculated with this material. The Indian and Bangladeshi vaccines had slightly different sequence modifications (Fig. 2), suggesting either two independent insertion events at the same point or an unstable insertion event during goat passage of the vaccine virus which resolved in two different ways. Other samples of GtVacc prepared in Kenya, or grown at Pirbright from samples sent from Kenya, did not have this insertion, showing that the insertion event occurred in India after the GtVacc was transferred to Kenya.

Isolates of other morbilliviruses have been found with additional bases, always in multiples of six and usually in the M-F UTR. A variant of peste des petits ruminants virus (PPRV) with an insertion of six bases in the F gene 5′ UTR was recently identified in China in 2013^28^. Similarly, several variants of measles virus (MV) have been found with a net insertion of six bases^29–31^. The mechanism of how these insertions and deletions occur is not yet clear, though it has been suggested from studies of MV genome variants that it is the result of errors of the viral polymerase when transcribing regions with extended homopolymers^30^.

Most of the regulatory elements in the virus genome sequences (promoter sequences, gene start and stop sequences) were highly conserved. A notable variation was in a group of Middle Eastern isolates from the 1980s and 1990s (RPV/Oman/79, Saudi/81, Yemen/81, Lebanon/82, Kuwait/83, Iraq/85, Turkey/92, Iran/94, Iran/95), where the otherwise conserved H-L intergenic region (CGT, 9197–9) had changed to CTT, the same sequence as all the other intergenic regions in the virus. This may have had an effect on transcription of mRNA encoding the viral RNA-dependent RNA polymerase (L protein), and it has been recorded that several members of this group showed particularly high virulence^32^.

Another notable sequence variation was the absence of a classical start codon for the F protein of a group of viruses found in sub-Saharan Africa in the period 1983–93. The start codon for the F protein is normally assumed to be that at 590–2 of the F gene transcript: this is immediately followed by sequence encoding a classic hydrophobic signal peptide and cleavage site^33^, followed by the highly conserved sequence at the start of the F2 peptide of the F protein^34^ (Supplementary Fig. S1). However, the genome sequences found in the viruses circulating in Nigeria in 1983, Egypt in 1984, Kenya in 1988–91 and Sudan in 1992–3 (Egypt/84, Kenya/Kajiado/88, Kenya/Kiambu/88, Kenya/Ngong/88, Kenya/Olentoko/89, Kenya/Suswa/88, Kenya/WPokot/86, Kenya/WPokot/89, Kenya/WPokot/91, Nigeria/Tambo/83, Nigeria/Yankari/buffalo/83, Sudan/93/RBsS, Sudan/Wakobu/92/RBS) have AUA (normally isoleucine) at this position instead of AUG. Interestingly, in these genomes there is no upstream AUG in the correct reading frame to give rise to the F protein, although all of these isolates (and only these isolates) have an AUG at 514–6, which is in the wrong reading frame (−1 relative to the F protein ORF). This contrasts with the F gene of RPV/Nigeria/Sokoto/1964, which has the even less efficiently used ACA codon at 590–2, but has an in-frame AUG codon just upstream at 545–7 (Supplementary Fig. S1). These data suggest that this group of viruses had to rely on abnormal translation or translation initiation in order to generate F protein.

The initiation of translation of the F protein from F gene mRNA is unusual in several morbilliviruses. The first RPV genomes sequenced^8,9^ had an additional in-frame AUG codon well upstream of the putative leader peptide sequence, at 320–322, and it was not clear which AUG was used for translation. Some, but not all, RPV isolates also have upstream AUGs which might give rise to F proteins with extended peptides before the signal peptide proper, e.g. India/Bison/89, SriLanka/87 and Korea/Fusan-B at 269–71, and the entire set of lapinised viruses, which have the first AUG at 152–4 of the F gene transcript, translation from which would give rise to a very extended F signal peptide, but would avoid initiation at the AUG at 561–3 (reading frame +1 relative to the F protein), also found in all the lapinised virus sequences. In the sequences presented here, about half the genomes have upstream AUGs that are in the wrong reading frame to give rise to the F protein; in addition to the African isolates mentioned above, RPVs Afghanistan/95, India/Ajmer/54, India/Bangalore/72, India/Bison/89, India/Bombay/54, India/HillBull, India/Hissar/53, India/Ranipet/73, India/Ranipet/80, Oman/79, Pakistan/85, Russia/89, Russia/Tuva/92, SriLanka/87, and Turkey/Pendik/49 all have an AUG codon at 91–3 (reading frame −1 relative to the F protein). In all these cases, as with the African viruses from late 1980s/1990s, F protein expression would require either a translational frameshift^35^ ocurring between the out-of-frame AUG and the coding sequence for the signal peptide, or leaky scanning to get past the incorrect AUG with, in some cases, translation initiation from an AUA codon in order to generate the F protein, despite the low efficiency with which this codon is used^36^. A third possibility is that the long 5’UTR sequence of the F gene transcript has the ability to direct the ribosome to start translation from a particular codon: work on MV^37^ showed that the long UTR directs translation initiation to the second available AUG in frame with the F protein ORF, ignoring the first.

The dependence on abnormal translation would be expected to lead to reduced expression of the F protein, which may be important for viral fitness. Although the RPV/Egypt/84 virus was recorded as being particularly mild^32^, there is no indication from the literature that all of the isolates with the variant start codon were particularly attenuated. Studies on MV^38^ and on canine distemper virus (CDV, another morbillivirus)^39^ showed that removing the long UTR in this region increased F protein expression. In the case of CDV it was shown that this also severely attenuated the virus, suggesting that limiting F protein expression is important for pathogenesis.

The 3′ ends of the genome and antigenome act as the promoters (RNA polymerase binding sites and sites of transcription initiation) for transcription of the antigenome and genome respectively and are referred to as the Genome Promoter (GP) and Antigenome Promoter (AGP). The GP also acts as the promoter for the transcription of viral mRNAs. The AGP was highly conserved across all RPV strains sequenced, with the first 18 bases of the genome completely conserved in all the isolates sequenced, as were 41 out of the first 50 bases. The GP is less conserved, with a variant base at position 5 and another at position 12, and only 34 conserved in the first 50 bases. The vaccine strains of both RPV and PPRV have a G at position 5 of the GP, while their virulent parents and most virulent strains have A, leading to the suggestion that this is an attenuating mutation^40,41^. However, we found a G in this position in the virulent viruses isolated in Russia in 1989 and 1992, and in the cattle-passaged Fusan parent of the Nakamura III lapinised virus^24^, suggesting that this mutation is not attenuating by itself, or that it can be compensated for by other mutations elsewhere in the genome.

We consistently observed small numbers of changes in the viral sequence during cell culture passage; it was possible to observe a change in sequence from one base, though a mixture, to a different base. These changes were, however, few in number, and usually silent: over 12 passages in cell culture, the RPV/Lap/AVRI strain showed only 11 consistent changes, and 8 of these were silent. The set of RBOK vaccine strain sequences also showed few differences, perhaps because they were already adapted to cell culture. Out of 12 positions showing variations in at least 3 samples, 7 were silent, or led to homologous changes in amino acid. All the other variations were in the F or H glycoproteins, and may reflect adaptation from the original bovine kidney cells^42^ to Vero cells (as used to grow the vaccine in later stages of the eradication programme).

### Copy-back RNAs in virus samples

**Figure 3:**
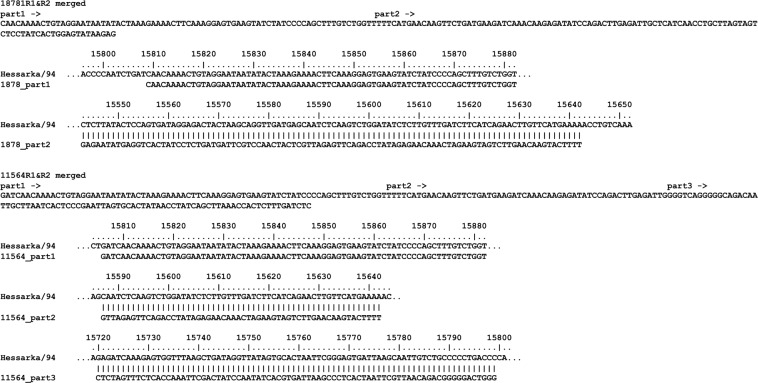
Examples of chimeric reads derived from copyback cDNA. Two cDNAs from the original RPV/Iran/Hessarka/94 library that were identified as mapping to more than one site were reconstructed by merging their respective R1 and R2 reads. The places on the genome from which these chimeric cDNAs were derived are shown. (**a**) Example of a simple copyback RNA where part of the RNA is transcribed from a genome sense template (exact match to the antigenome) while the second part is the complement to the antigenome sequence. (**b**) An example of a chimeric RNA that maps to three different locations, suggesting the polymerase swapped templates as in (**a**), so that 11564_part2 is the complement to the antigenome, and then jumped to another part of the template, but on the same strand, so that 11564_part3 is also the complement to the antigenome.

**Figure 4:**
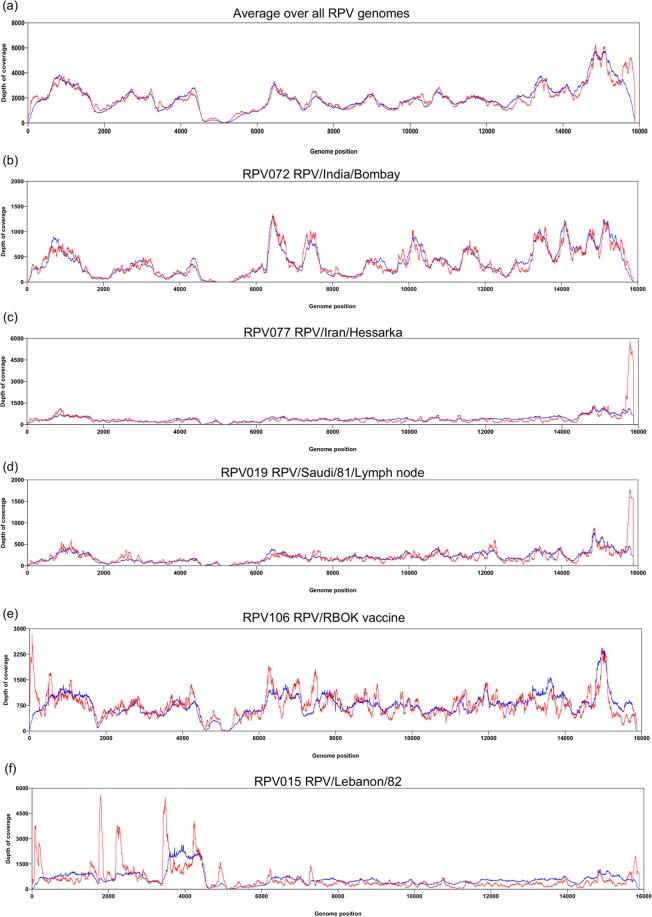
Presence of defective interfering particles in virus preparations revealed by NGS. Reads derived from chimeric RNAs were extracted and the depth of coverage for each genome determined as for all reads. The average coverage for chimeric reads was normalised by multiplying by the ratio of total number of mapping reads to total number of mapping chimeric reads. The depth of coverage of the genome by all matching reads (blue) or chimeric reads (red) is shown. (**a**) The average coverage taken over all genomes sequenced; (**b**) a preparation of challenge virus labelled India/Bombay; (**c**) a cell culture-grown preparation of RPV from Iran; (**d**) a lymph node sample from an animal infected with RPV/Saudi/81; (**e**) a preparation of the standard RPV vaccine; (**f**) a preparation of cell-culture-grown RPV/Lebanon/82.

The NGS datasets revealed that chimeric RNAs, that is RNA transcripts that mapped to more than one part of the genome, were found in all preparations of RPV, whether cell cultured virus or infected tissue. These chimeric RNAs appeared to arise from copy-back events, as the supplementary alignment was always on the opposite strand to the primary alignment (for examples, see Fig. 3). The fraction of RPV-derived cDNAs in the sequencing libraries that had supplementary alignments varied from 0.9% to 16.4% (mean = 9.4%, s.d. = 2.7%), and this fraction did not differ significantly between cell cultured virus and infected tissues. The chimeric RNAs appeared to be transcribed from all parts of the genome, the amount of chimeric RPV RNA closely following the overall pattern of RPV RNA in the samples (Fig. 4). However, some samples showed strong peaks of chimeric RNAs at specific positions along the genome, notably at the AGP (compare Fig. 4a,c,d with Fig. 4b), suggesting that these samples contained a copy-back defective interfering particle (DI) that included the AGP at each end and was thus replicated efficiently. Preparations containing a DI based on the GP could also be identified (Fig. 4e,f). Some preparations also showed strong peaks at internal points along the genome (Fig. 4f); these regions do not contain promoters and so copy-back RNAs containing these sequences should not be amplified, and further investigation will be required to identify the exact nature of the chimeric RNAs in these isolates, and whether this pattern is seen in related viruses such as measles virus.

Copy-back DIs have long been known to be produced during the replication of RNA viruses, and are normally seen in virus preparations that have been passaged in cell culture at too high a multiplicity of infection (m.o.i.)^43,44^. Most of the samples found to contain the signature of a replicable copy-back DI were indeed from cell culture passaged virus; the exceptions were the tissue samples taken from an animal infected with RPV/Saudi/81 (Fig. 4d), adding RPV to the list of viruses including influenza virus^45^, dengue virus^46^ and West Nile virus^47^ for which DIs have been found in natural infection.

**Figure 5:**
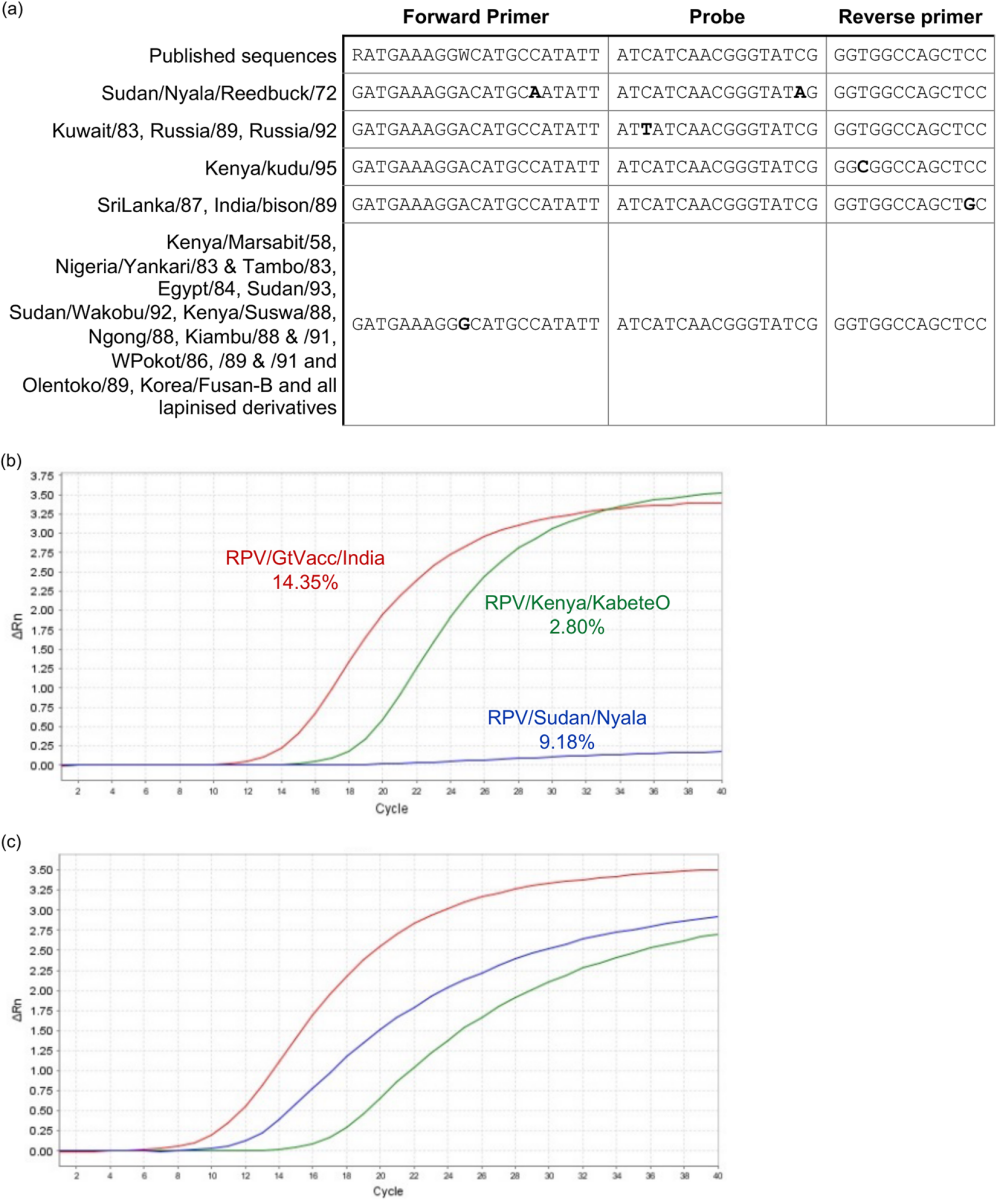
RPV isolates with variant sequences at the target sites of the RPV RT-qPCR. (**a**) The published sequences for the forward and reverse primers and the probe sequence for the RT-qPCR assay of Carrillo *et al*. (2010) are shown, along with variants identified in the genomes presented here. Variant bases are shown in bold. (**b**,**c**) Amplification curves for RT-qPCR of samples of those RPV isolates where deviation from the probe/primer sequences had an observable effect, compared to isolates with sequences matching the primers and probe. Numbers next to isolate names show the percent of the reads in the corresponding sequence library that mapped to RPV genome. (**b**) Amplification curves observed with RPV/Sudan/Nyala/Reedbuck/72 (blue) compared to RPV/GtVacc/India/Edwards/spl (red) and RPV/Kenya/KabeteO (green). (**c**) Amplification curves of RPV/Kuwait/83 (blue) and RPV/Russia/89 (green) compared to RPV/Nigeria/Tambo/83 (red).

The only published RT-qPCR assay for RPV^18^ was developed after the virus was declared eradicated, and so was not subject to the kind of global testing undergone by the simple RT-PCR assays in use during the control and eradication programme^6^. It was therefore useful to assess this assay against the large set of RPV isolates in this study. About a third of isolates had a G at position 10 of the forward primer instead of the published A/T (Fig. 5a). We also found mismatches in the reverse primer for RPV/Kenya/kudu/95 and RPV/SriLanka/87 and in the probe for RPV/Kuwait/83, Russia/89 and Russia/92, and for RPV/Sudan/Nyala/Reedbuck/85 (Fig. 5a). This last mismatch, located close to the 3′ end of the probe, was the only one to have a major effect on the assay (Fig. 5b), possibly because it was coupled in this virus with a mismatch near the 3′ end of the forward primer; the reaction was obviously much less efficient, giving a very high Ct despite the NGS sequencing library having a high content of RPV sequence; this deviation from the consensus did not completely prevent the detection of this isolate of RPV, but did reduce the sensitivity of the assay when that isolate was the target. The viruses which had a mismatch closer to the 5′ end of the probe (RPV/Kuwait/83, Russia/89 and Russia/92) also showed lower efficiency in the RT-qPCR (decreased slope of the amplification plots) (Fig. 5c), but the differences here were relatively minor. All other isolates showed essentially normal amplification efficiency (not shown) despite a difference of one base in the forward or reverse primer. This assay has been adopted at Pirbright in its role as OIE Reference Laboratory for Rinderpest and FAO World Reference Laboratory for Ruminant Morbilliviruses, albeit with modification to improve probe binding (Methods). The assay clearly works for almost all virus isolates, but the possibility of a naturally occurring variant having decreased detection efficiency should be borne in mind.

### Phylogenetic analysis of the RPV genomes

The genome sequence was partitioned into coding sequence (CDS) and untranslated regions (UTR), and the CDS further partitioned by codon position, giving a total of 25 partitions which were then grouped using *PartitionFinder2*^48^ into 6 groups of partitions, the members of each group having essentially the same parameters for the best fit evolutionary model (see Methods for details). The partitions, and the groups they were assigned to, are given in Tables 1 and 2. The UTRs were mostly grouped together, the consistent exception being UTR4, encompassing the long GC-rich region between the M protein CDS and the F protein CDS; in addition to having a high C/G content (Fig. 1), this region was notable for a very strong strand-specific A/T bias, with an A:T ratio of 4.8 for the antigenome strand, compared to ~1 for the other UTRs (Group 1) and for the groups containing CDS codon positions 2 and 3 (Groups 4 and 5); the CDS codon positions 1 showed the slight bias towards A over T (A:T = 1.8) that has previously been reported for a large number of eukaryotic CDS^49^.

**Table 1:** Regions of the RPV genome used in partitioning the sequence.

	Start	End	Region
UTR1	1	107	GP
CDS1	108	1685	N protein
UTR2	1686	1806	N/P i.g.
CDS2	1807	3330	P protein
UTR3	3331	3437	P/M i.g.
CDS3	3438	4445	M protein
UTR4	4446	5412	M/F i.g.
CDS4	5413	7098	F protein
UTR5	7099	7258	F/H i.g.
CDS5	7259	9088	H protein
UTR6	9089	9221	H/L i.g.
CDS6	9222	15773	L protein
UTR7	15774	15882	AGP

Each CDS is the coding sequence for the given protein; note that the P protein CDS also functions to encode the C and V non-structural proteins. For analysis, each CDS was further subdivided by codon position, giving (e.g.) CDS1_pos1, CDS1_pos2, CDS1_pos3, etc. The untranslated regions (UTR) consisted of the Genome Promoter (GP) and AntiGenome Promoter (AGP) regions at the ends of the genome and the non-coding sequences from one CDS to the next, including the intergenic (i.g.) sequence motifs that function as transcription stop/transcription start signals.

**Table 2:** Partitions of the RPV genome used in phylogenetic analysis.

Partition group	Group members
1	UTR1, UTR2, UTR3, UTR5, UTR7, CDS2_pos3
2	UTR4
3	CDS1_pos1, CDS3_pos1, CDS4_pos1, CDS6_pos1
4	CDS2_pos1, CDS2_pos2, CDS5_pos1
5	CDS1_pos2, CDS3_pos2, CDS4_pos2, CDS5_pos2, CDS6_pos2
6	CDS1_pos3, CDS3_pos3, CDS4_pos3, CDS5_pos3, CDS6_pos3, UTR6

*PartitionFinder2* was used to group the available functional partitions (Table 1) until no further improvement was observed. These partitions were used for both maximum likelihood and Bayesian phylogenetic analysis.

A notable exception to the partitioning of codon positions 1 and 2 was found for CDS2. CDS2 encodes the P protein (a structural protein which links the nucleocapsid protein (N) to the viral polymerase (L)) and also two non-structural proteins, C and V. The C protein is encoded in an alternate reading frame such that codon position 2 for the P protein open reading frame is codon position 1 for the C protein open reading frame; these overlapping reading frames are probably the reason why CDS2_pos1 and CDS2_pos2 group together, and separately from most of the other CDS position 1s.

**Figure 6:**
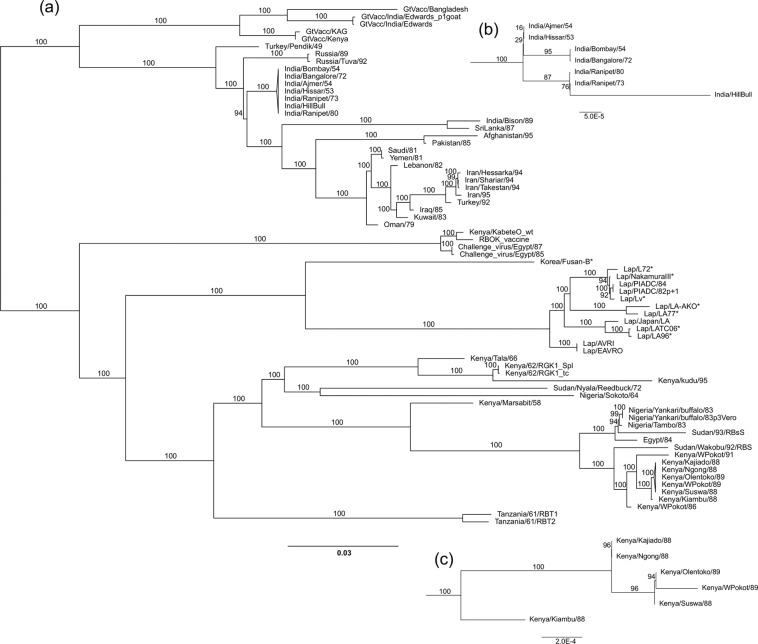
Phylogenetic analysis of RPV isolates. The evolutionary relationships between the RPV isolates were inferred from their sequences by maximum likelihood as described in Methods. The analysis was based on the unique genome sequences determined in this study plus 8 Asian isolates previously published (*). (**a**) The maximum likelihood tree in which the robustness of the resultant branches was assessed by the percentage of replicate bootstrapped trees (1000 replicates) in which that grouping of taxa occurred. The scale bar calibrates the evolutionary distances (branch lengths) in substitutions per site. The two groups of very closely related isolates headed by India/Ranipet/82 and Kenya/Kajiado/88 have been drawn as single groups; enlargements of these subclades are shown as inserts (**b**,**c**) respectively.

For the phylogenetic analysis, duplicate sequences were removed and independent samples of the same isolate replaced with a consensus sequence; 8 Asian RPV genome sequences that have been previously published^24,26,27^ or simply deposited in the public sequence databases were included for comparison. The final alignment included 70 genomes, which were analysed by both maximum likelihood (ML) (Fig. 6) and Bayesian methods (Supplementary Fig. S2) in order to avoid any errors linked to the known weaknesses of either method^50^. In fact, the phylogenetic trees produced by the two methods differ only in minor details of the relative placement of the set of very closely related Indian sequences. In both cases, the tree branches were very strongly supported by the estimates of robustness, i.e. the results of the bootstrap (ML tree) or the posterior probability values (Bayesian tree).

The phylogenetic trees produced by both analyses are unrooted (i.e. no molecular clock is suggested), although for clarity in identifying the RPV isolate at each tip we have displayed them in the style of rooted trees. The root of the RPV tree was determined by carrying out phylogenetic analyses on the same set of RPV genomes but with the inclusion of a MV genome sequence to act as an outgroup (Supplementary Fig. S3), and by assuming that the ancestral node closest to the MV sequence would be the root of the RPV tree. This placed the root on the section of the tree between the RPV/KabeteO clade and that containing the goat-adapted vaccines, the two clades derived from the oldest isolates of RPV.

As expected, there was a clear differentiation between isolates from countries in Asia and the Middle East and those from countries in Africa (Fig. 6). Previous studies of the evolutionary relationships of different RPV isolates^7,51^ divided the viruses into three lineages, one covering all isolates from Asia, the Middle and Near East, and two containing all the isolates from Africa (Africa 1 and Africa 2). These analyses were based on a relatively short (322 bases) stretch of the F CDS, and many of the sequences used in those analyses were from PCR products obtained from diagnostic samples; the associated viruses were not isolated and were therefore not available to us for full genome sequencing. Africa 1 contained (among others) RPVs Egypt/84, Sudan/92 and the Kenyan isolates from 1988, 1989 and 1991, which are all still closely linked when their full genome sequences are analysed. Africa 2 contained (among others) Tanzania/61/RBT1, Kenya/62/RGK1, Kenya/Kudu/93 and Nigeria/Sokoto/64; however, the full genome sequence of these isolates shows that their most recent common ancestor is very distant from all of the other available sequences. It may not be reasonable to consider these viruses a single clade or lineage, especially as Tanzania/61/RBT1 clearly came from a branch that separated off from the others before the split between “Africa 1” and the rest.

Historically, RPV is thought to have made a single incursion into sub-Saharan Africa in 1887^52^, and spread rapidly over most of the continent. It was always a significant puzzle as to why there were two distinct clades in Africa, or three if one includes the clade containing RPV/KabeteO and its derivatives, given that all African RPV isolates are thought to come from this single epizootic. The new data suggest that, based on their evolutionary distance from each other, the African isolates fall into at least 5 clades (Tanzania/61/RBT1&2; Kenya/62/RGK1, Kenya/Kudu/93 and Kenya/Tala/66; Sudan/Nyala/Reedbuck/85; Nigeria/Sokoto/64; the rest) which differ from each other by more than the distances separating any of the available Asian isolates. Alternatively, they can be seen as a single large clade, with multiple branches that probably represent the isolation of the virus at very different times and in different geographical areas, a pattern that is more in conformity with the available historical evidence.

For comparison with previous publications, we generated a similar phylogenetic tree using all the available 322-base sections of the RPV F gene (Supplementary Fig. S4), including those derived from diagnostic samples. It is clear from these data that, while there is a strong clade containing many mostly East African samples, the Nigeria/58, Tanzania/61 and Nigeria/Sokoto/64 isolates each form a separate branch, as does the group of Kenyan isolates including Kenya/Tala/66 and Kenya/62/RGK1. Because of the limited amount of data for each isolate, the support values for many of the branches are not as strong as those for the full genome sequences, but the overall pattern is very similar.

In the whole genome analyses, the clade containing the Kabete ‘O’ isolate and related viruses clustered with the other African isolates, although on a distinct branch, reflecting that the parent of these viruses was originally isolated in Kenya in 1910. The Kabete ‘O’ challenge strain, from which Plowright derived his vaccine by repeated passage in BK cells^53^, was originally maintained by cattle passage for use as a standard challenge virus^54^, and this virus was clearly shared with other countries, as the challenge virus in use in Egypt in the 1980s was a variant of the Kenyan Kabete’O’ virus, and not a wild type virus from Egypt. An unexpected finding was that the entire group of lapinised viruses related to the original Nakamura III vaccine^55^, including the Korea/Fusan-B which is thought to be the virus from which Nakamura derived his vaccine^24^, was clustered with the African viruses. This is not a reflection of how the figure is drawn, but of the fact that the evolutionary distance from the Nakamura/Fusan clade to the African viruses, as estimated by both ML and Bayesian methods, is less than the distance from the RPV/KabeteO clade to the rest of the African viruses. This finding was unexpected as there is no history suggesting a link between scientists working in Africa and the Japanese scientists working at Fusan in Korea at the time (approximately 1934–8) when Nakamura was beginning his work on adapting RPV to rabbits^56^. Unfortunately, the origin of the virus used in those studies was not given, nor was it referred to as anything other than “Laboratory Strain”^56^, although Nakamura himself refers to it in a later work as the “laboratory “O” strain”^57^, reminiscent of the name (Kabete ‘O’) of the strain used in Kenya at that time. Further research may clarify the exact origin of the virus used in Korea for these studies. It is interesting to note that the lapinised RPV held at the EAVRO in Kenya (and at some time transferred to AVRI in the UK for the preparation of anti-RPV sera for use in diagnosis) branches off the lapinised virus line before the extra adaptation steps that lead to the current set of Japanese/Korean vaccines. This virus may thus represent something closer to the original Nakamura III virus^56^ than the sample sequenced under that name, which had undergone many further passages in rabbits since the original vaccine was created.

The goat-adapted viruses, originally developed for use as a vaccine during the late 1920s in India^58^, form a distinct clade which, as is the case with the Kabete ‘O’/RBOK-vaccine viruses, probably reflects both the long time since their isolation in the wild and the artificial way in which the virus was maintained. An interesting observation in this group of sequences is that the so-called Kabete-Adapted Goat (KAG) vaccine virus, originally reported as having been developed from the Kabete ‘O’ wild type virus^59^, is clearly not related to RPV/KabeteO at all but is closely related to the Indian goat-adapted viruses. The sequence of the RPV/KAG virus was the same in all the samples sequenced, including unopened vials prepared at EAVRO in Kenya. The attenuation of the Kabete ‘O’ virus over 250 passages in goats is on record^60,61^. These data, however, suggest that at some point the Edwards goat-adapted virus, sent to Kenya in 1936^62^ was switched with the African virus being passaged there in goats and, in the absence of any way of distinguishing strains at that time, this mistake was then propagated.

The sequences of a large selection of Indian samples of RPV, recorded as having been prepared as challenge virus from samples taken in different places at different times (See Supplementary Table S1), were essentially identical. The sample labelled “Hill Bull” is a sample of one of the standard challenge virus used at Mukteswar in India for many decades; though it is unknown exactly which one is represented by RPV/India/HillBull. While it was reasonable to expect the Ajmer, Hissar and Bombay isolates, all from around 1953–4, to be similar to each other, the Bangalore isolate of 1972 and those from Ranipet in 1973 and 1980 appear to be essentially identical to those viruses from 20–30 years previously. It is possible that RPV in India had become completely stable over a long period, having fully adapted to the local hosts, and showing minimal sequence drift from 1950–80; however this does not accord with the continuous genetic drift seen in other RPV isolates from the same region and time, such as the India/bison/89 and Sri Lanka/87 isolates. It is more likely that an established challenge strain was repeatedly re-isolated from cattle and relabelled as a new challenge strain. Despite the intensive work on RPV carried out over many decades in India, primarily at Mukteswar, we have relatively few genome sequences from this part of the world. It will be useful if any viruses still being held in India are similarly sequenced before being destroyed.

In summary, we have sequenced, and now destroyed, almost all the RPV held at the Pirbright Institute. The full genome sequences provide evidence supporting a single entry of the virus into sub-Saharan Africa and its expansion into multiple subclades. Further bioinformatic analyses may reveal more detailed information about the growth and evolution of RPV.

## Materials and Methods

### Viruses

All the virus samples used in these studies were from the archive at the Pirbright Institute. The complete list of RPV isolates sequenced, the methods of RNA extraction and purification, the method used to prepare the sequencing library and the fraction of RNA mapping to RPV in each case is given in Supplementary Table S1, along with the sample history.

### RNA extraction

Several methods were used to extract RNA from samples of cultured virus or from samples of tissue from infected animals. Extraction with phenol-based reagents followed by ion-exchange spin-column purification was effective for cell culture supernatants containing cultured virus; for many tissue samples, this method gave RNA that clearly contained an inhibitor of the RT or PCR step, as shown by an improved response in RT-qPCR after sample dilution. Extraction with the Kingfisher automated magnetic bead system gave reproducibly cleaner results, although lower yield, and was adopted as the standard method by the end of the project.

TRIzol LS was used to dissolve cell culture samples, followed by RNA extraction using the Direct-zol RNA Miniprep kit or phase separation using the TRIzol protocol, when the RNA was either precipitated directly using isopropanol, or extracted from the aqueous layer using the Zymo RNA Clean & Concentrator-5 kit. For extraction of RNA from freeze-dried tissue, the sample was resuspended directly in 1 ml of TRIzol reagent and extracted using one of the methods above. Automated extraction of RNA was carried out using the LSI MagVet kit on a Kingfisher Flex Purification System.

### Preliminary screening using RT-qPCR

The level of RPV-specific RNA in samples was estimated using a variation on the previously published RT-qPCR assay targeting the L gene^18^ in which the probe contained a minor groove binder and non-fluorescent quencher to improve its effective melting temperature. Reactions were performed using 3 µl of sample RNA in a 20 µl reaction volume on an AB7500 fast real-time PCR instrument: reverse transcription at 50 °C for 15 min, Taq activation at 95 °C for 20 s then 40 amplification cycles of 95 °C for 3 s and 60 °C for 30 s.

### NGS library preparation

Sequencing libraries were prepared using either transposon-based fragmentation of cDNA (Nextera XT DNA Library Prep kit, Illumina) or single primer isothermal amplification (SPIA) (Trio RNA-Seq kit, NuGEN); in each case the reactions were carried out according to the manufacturer’s instructions.

For library preparation using the Nextera kit, first strand cDNA was generated from total RNA (0.4–4 µg depending on concentration) using random hexamer primers and SuperScript III reverse transcriptase according to the manufacturer’s protocol. RNA was then digested at 37 °C for 20 min with 2U RNase H and double stranded cDNA synthesised using NEBNext Second Strand Synthesis enzyme mix and reaction buffer in a final volume of 80 µl at 16 °C for 2.5 h. Double stranded cDNA was purified using the Illustra GFX DNA purification kit. The concentration of cDNA was assessed using the Qubit dsDNA HS assay kit and adjusted to a final concentration of 0.2 ng/µl. Libraries were generated from 1 ng cDNA using the Nextera XT DNA Library Prep kit.

For library preparation using SPIA, RNA was quantified using the Qubit RNA HS quantification kit and 50 ng total RNA was used for library preparation with the Trio RNA-Seq kit. After amplification, enzymatic fragmentation and library construction, host rRNA sequences were depleted as directed by the kit manufacturer (Trio RNA-Seq kit, NuGen).

For all libraries, paired-end read sequencing was carried out using the Illumina MiSeq platform and version 2 reagents.

### Analysis of NGS data

All NGS datasets were analysed with a custom script in which the data was first quality trimmed using *Sickle*^63^ and then mapped to the sequence of RPV/KabeteO (Accession numbers NC_006296/X98291) using *bowtie2*^21^ and *bwa-mem*^22^. In each case, duplicates were removed from the reads that mapped to the bait sequence using the *SAMtools* package^64^ and the consensus sequence was determined using the same package. The two consensus sequences were then compared, any disagreements resolved by manual inspection of the read data, and the two merged into a single consensus. Regions at the genome ends or in the M-F GC-rich region that were not determined by the NGS data were filled by RACE (genome ends) or a specific GC-rich PCR protocol.

### Amplification and sequencing of the GC-rich region

The GC-rich region was amplified in two fragments using the primer pairs GC_F2/Frag3R and Frag4F/GC_R2 (sequences of all named primers are given in Supplementary Table 2). cDNA template was prepared using Superscript III according to the manufacturer’s instructions. PCR was carried out using KAPA HiFi reaction mix (Roche) with 10pmol each of forward and reverse primer and 2 µl of first strand cDNA template (50 µl final volume). The PCR cycling conditions were: 95 °C for 5 min, then 40 cycles of 98 °C for 20 s, 60 °C or 54 °C (for the GC_F2/Frag3R and Frag4F/R2 primer pairs, respectively) for 15 s, 72 °C for 1 min, and a final elongation step of 72 °C for 1 min. In cases where the primers above did not result in amplification, isolate-specific primers with high melting temperatures were designed based on the next generation sequencing data and PCR carried out as above but using a combined annealing/extension step of 68 °C for 45 s.

In all cases, PCR products were purified using the Illustra GFX DNA purification kit, according to manufacturer’s instructions. The purified PCR product was sequenced in both directions using the BigDye Terminator v3.1 reagents with the addition of dGTP to 1 µM. The cycle sequencing conditions were: denaturation at 96 °C for 1 min followed by 30 cycles of 96 °C for 10 s, 50 °C for 5 s and 60 °C for 4 min.

### Rapid amplification of sequence ends (RACE)

To amplify the 5′ end of the genome and/or antigenome in order to determine the terminal sequences of isolates, a variant of our previously published RACE protocol^65^ was used. RPV-specific cDNA was prepared using SuperScript III and 2pmol each of RACE2, RACE3, RACE5 and RACE6 primers in a final volume of 20 µl. After incubation with 2U RNase H and 50U RNase 1 _f_ (NEB) at 37 °C for 60 min, single-stranded cDNA was purified using the Qiagen DyeEx 2.0 spin column purification kit. Purified cDNA (10 µl) was tailed in a 20 µl reaction containing 10U terminal deoxynucleotide transferase (TdT), 0.1 mM dATP and 0.5X complete SuperScript III reaction buffer; the reaction was incubated at 37 °C for 5 min followed by inactivation of TdT at 70 °C for 10 min.

The 5′ end of the genome was amplified in a 50 µl reaction containing 1X KOD hot start master mix (Merck Millipore), 15pmol each of Q1 and RACE4a primers, 0.5pmol QT primer and 2 µl tailed cDNA. Cycling conditions were: 95 °C for 2 min 20 s, 33 °C for 15 s, 70 °C for 15 s, followed by 40 cycles of 95 °C for 20 s, 53 °C for 10 s and 70 °C for 15 s. For the 5′ end of the antigenome, a hemi-nested PCR protocol was used in which the first stage was as for the 5′ end of the genome, except for the use of RACE1 primer instead of RACE4a. The product of this reaction was dominated by amplicons derived from the 5′ end of the N gene mRNA transcript, so the 5′ end of the antigenome was then amplified in a second PCR using 15pmol each Q1 and M13RACE7c primers and 0.5 µl 1^st^ round PCR product as template (50 µl final volume). Cycling conditions were: 95 °C for 2 min 20 s, followed by 40 cycles of 95 °C for 20 s, 63 °C for 10 s and 70 °C for 15 s. PCR products were purified as above and sequenced using RACE4a for the genome 5′ end and the standard M13 forward primer for the antigenome 5′ end. All Sanger sequencing was carried out on an Applied Biosystems 3730 DNA Analyser.

### Phylogenetic data analysis

Where an isolate had been sequenced from more than one sample, only the consensus sequence was used for phylogenetic analysis. Sequences of the same isolate after sequential passage were also not included. In addition to 62 sequences from this study, 8 published full length RPV genomes were included: cattle-passaged RPV/Korea/Fusan-B (AB547189) and the lapinised derivative RPV/Lap/NakamuraIII (AB547190)^24^; three further lapinised/avianised derivatives of Nakamura III namely RPV/Lap/L72 (JN234008), RPV/Lap/LA77 (JN234009) and RPV/Lap/LA96 (JN234010)^26^; an additional lapinised/avianised strain, RPV/Lap/LATC06 (GU168576)^25^; the current lapinised/avianised RPV vaccine kept in Japan, RPV/Lap/LA-AKO (LC057619)^27^; an unpublished sequence of a lapinised strain from the University of Tokyo, RPV/Lap/Lv (LC168749). We did not include our previously published sequences for the RBOK vaccine strain and the Kabete ‘O’ wildtype virus^8,9^ as these viruses were included in the set of genome sequences determined in this study and those sequences would be more reliable than those originally determined from cDNA libraries.

The optimal partitioning of the sequences was determined using PartitionFinder2^48^. Optimisation was restricted to the most general models, the General Time Reversible (GTR) model with or without a gamma-distributed set of variable rates (+G) and a fraction of the sequence that was completely invariant (+I). The GTR + G + I model produced, overall, the better fit as judged by the Bayesian Information Criterion (BIC) values. This model was used for all subsequent analyses.

The ML tree was determined using RAxML^66^. The program was run 3 times, each time using 20 random trees as starting points, and the best fit tree taken from these results. Bootstrap support values were obtained from 1000 bootstraps using RAxML’s rapid bootstrapping algorithm. The best fit tree was also determined by Bayesian methods using MrBayes^67^. The default priors were used throughout, and the search was run for 1,000,000 generations. Tree figures were prepared with FigTree v1.4.4.

## Supplementary information

Supplementary Table.

Supplementary Information.

## Data Availability

All the genome sequences resulting from this study are available in the public sequence databases; the relevant accession numbers are listed in Supplementary Table S1.
