# Supplementary material for: Full genome sequencing of archived wild type and vaccine rinderpest virus isolates prior to their destruction
Source: Sci Rep. 2020 Apr 16;10:6563. doi: 10.1038/s41598-020-63707-z (PMC7162898; doi:10.1038/s41598-020-63707-z)
Supplement: Supplementary file 2 — Supplementary Information. [file 41598_2020_63707_MOESM2_ESM.pdf]

Full genome sequencing of archived wild type and vaccine rinderpest virus isolates prior to their destruction.

Simon King, Paulina Rajko-Nenow, Honorata M Ropiak, Paolo Ribeca, Carrie Batten, Michael D Baron

The Pirbright Institute, Ash Road, Pirbright, Surrey GU24 0NF, UK

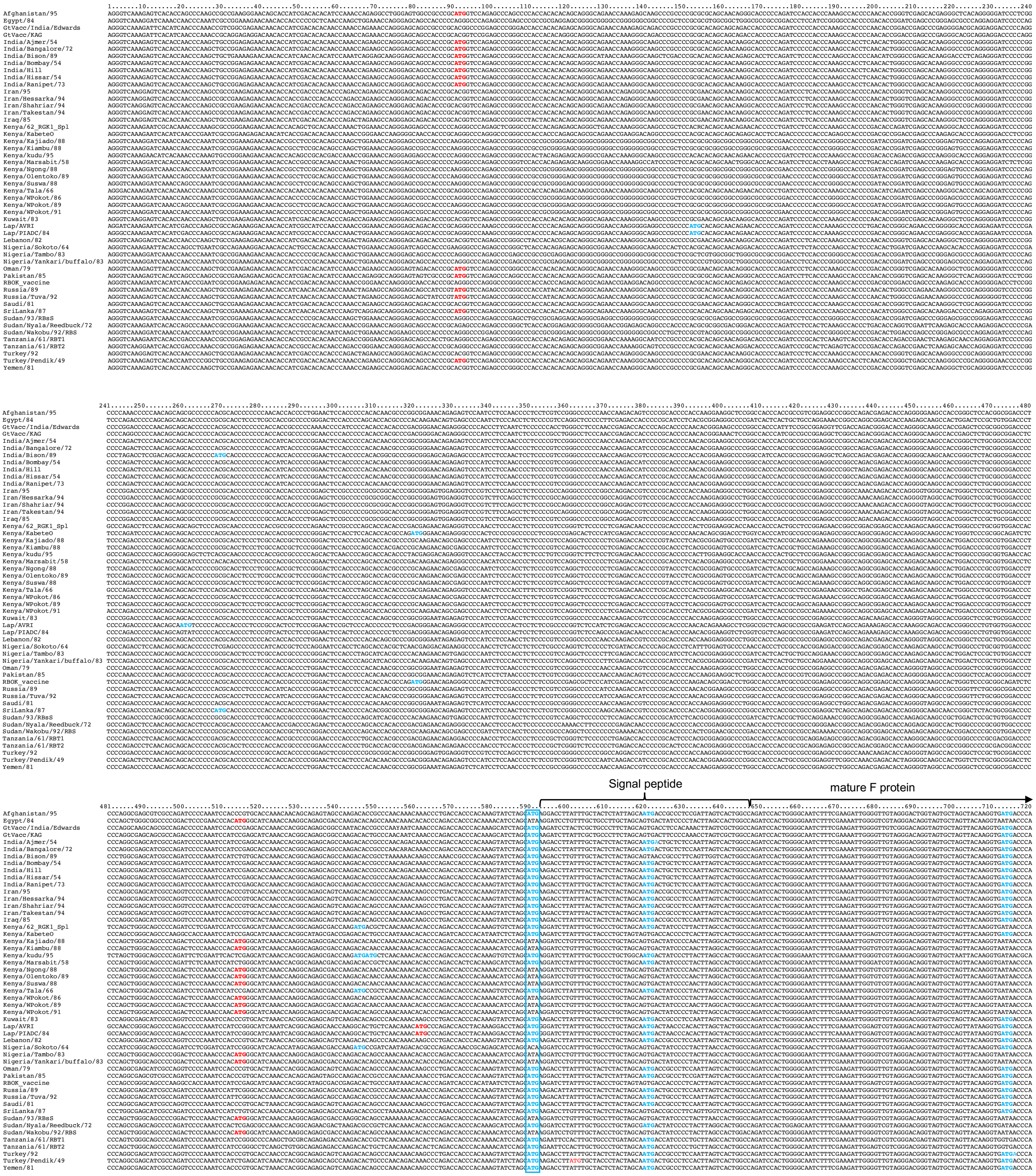

Figure S1 Alternate start codon usage in the RPV F gene. The figure shows the first 720 bases of the F gene of a representative set of RPV genomes. All ATG start codons are highlighted in **bold**. ATG codons that are in-frame with the F protein open reading frame are highlighted in **blue** and ATG codons that are in the wrong reading frame are highlighted in **red**. The position of the most probable start codon is outlined in blue. The position of the coding sequence for the F protein signal peptide and the mature F protein sequence is indicated.

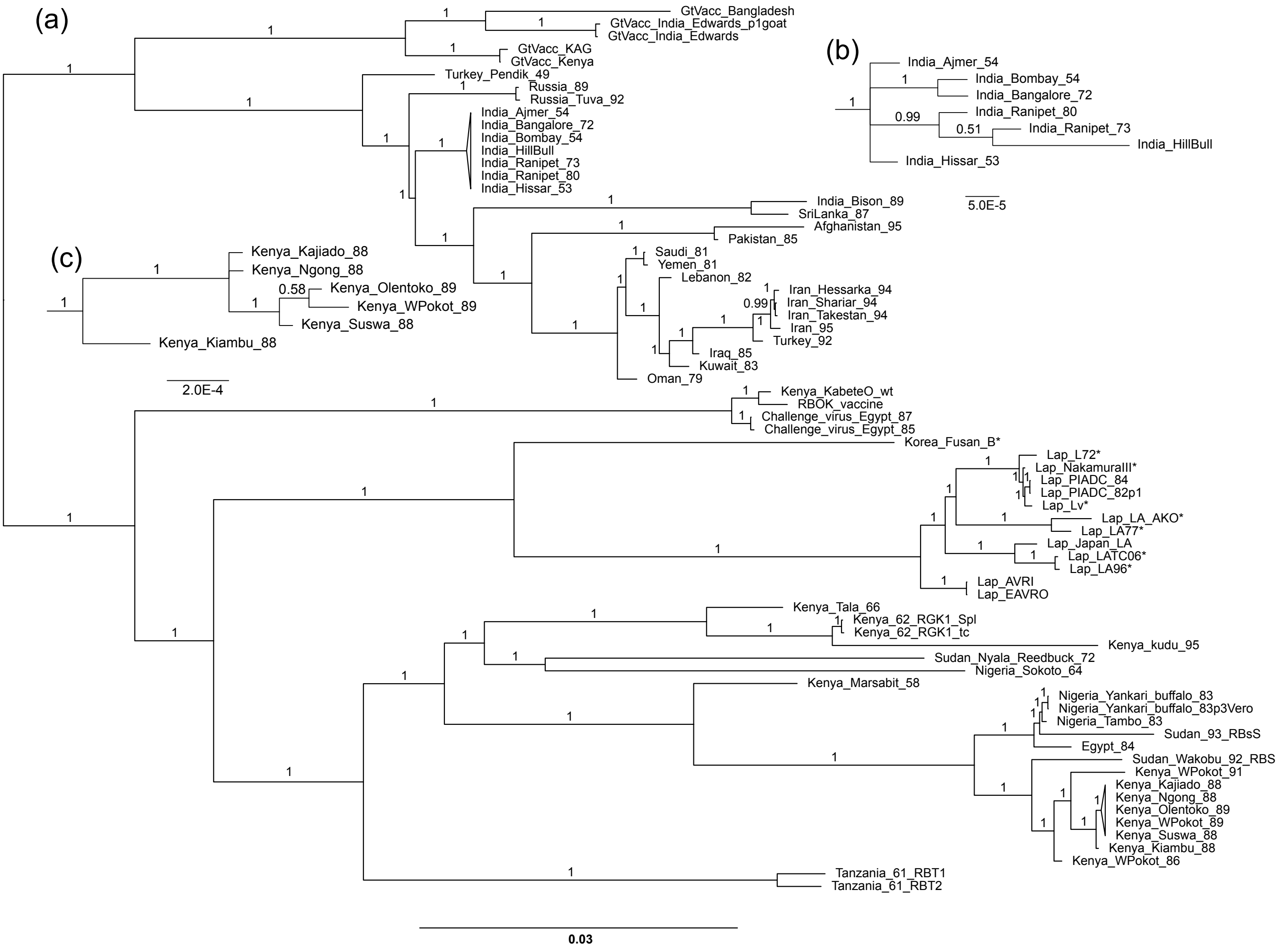

Figure S2 Phylogenetic analysis of RPV isolates. The evolutionary relationships between the RPV isolates were inferred from their sequences by Bayesian inference as described in Methods. The analysis was based on the unique genome sequences determined in this study plus 8 Asian isolates previously published (*). (a) shows the consensus tree after 1,000,000 generations, with each branch labelled with the posterior probability of that branch. The scale bar calibrates the evolutionary distance between isolates (branch lengths) in substitutions per site. The two groups of very closely related isolates headed by India/Ajmer/54 and Kenya/Kajiado/88 have each been drawn as a single group; enlargements of these subclades are shown in inserts (b) and (c) respectively. Note that, in order to comply with restrictions of the software used for Bayesian analysis (*MrBayes*), we have had to slightly alter the names of the isolates, notably the replacement of the more normal '/' with '_'

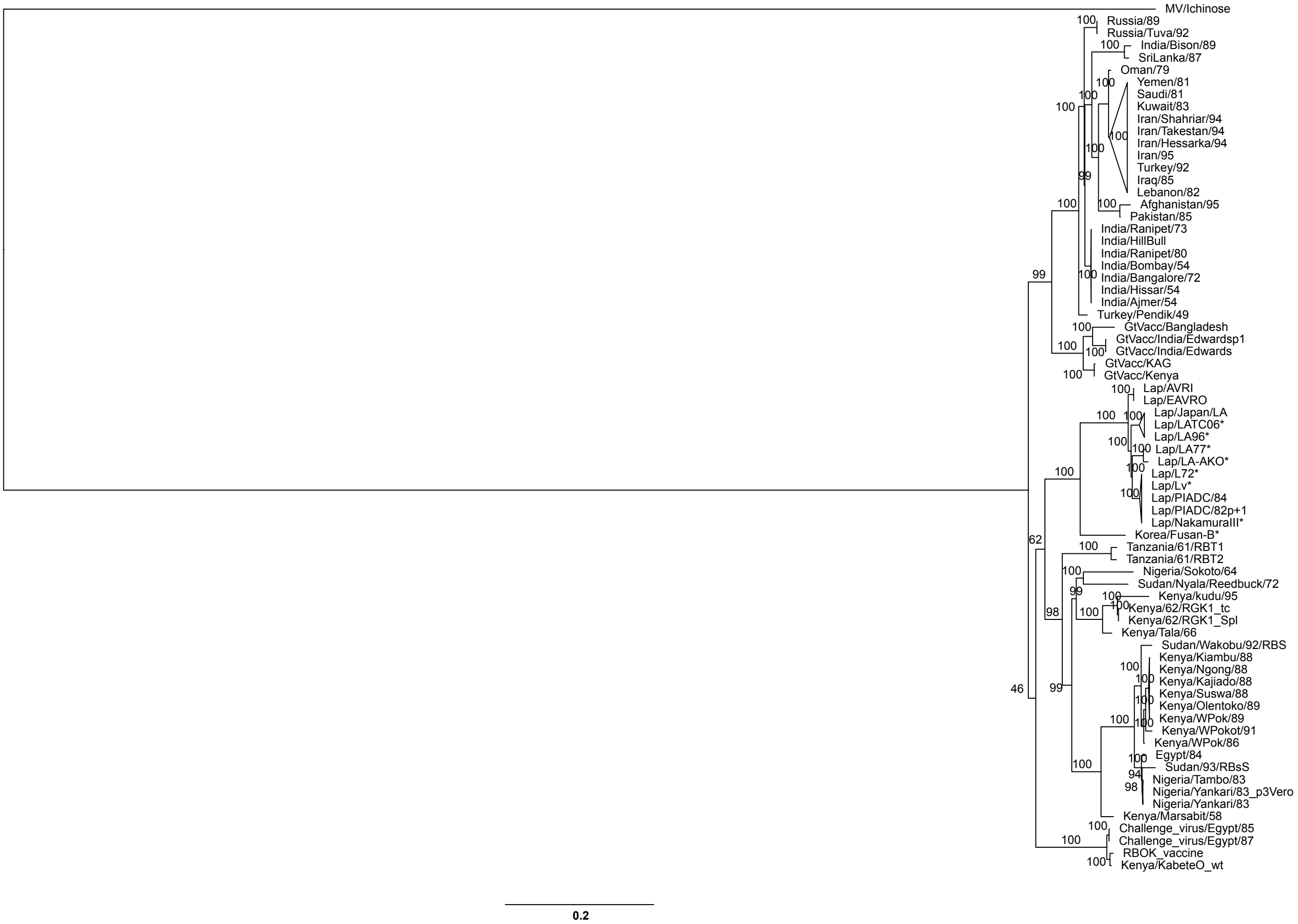

Figure S3 Identification of root of RPV tree using MV as an outgroup The evolutionary relationships between the virus isolates were inferred from their sequences by maximum likelihood as described in Methods. The analysis was based on the unique genome sequences determined in this study plus 8 Asian isolates previously published (*) plus one MV genome (Measles virus Ichinose-Vero, Accession # AB032167). The maximum likelihood tree is shown in which the robustness of the resultant branches was assessed by the percentage of replicate bootstrapped trees (1000 replicates) in which that grouping of taxa occurred. Because of the heavy compression of the RPV sequences to the right of the figure, various groups with 100% support have been collapsed to improve legibility. The scale bar calibrates the the evolutionary distances (branch lengths) in substitutions per site.

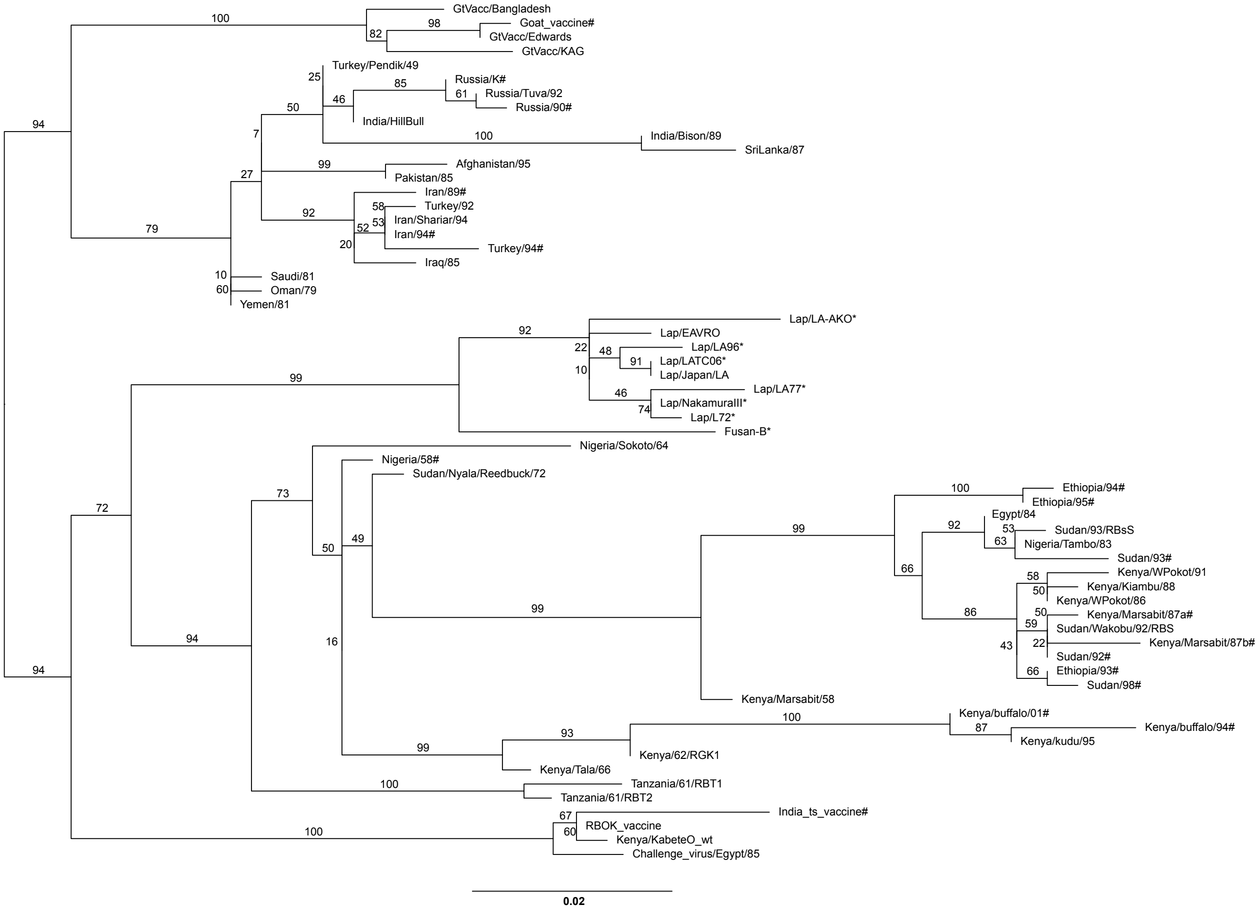

Figure S4 Phylogenetic analysis of RPV isolates based on sequences of F gene PCR products. The evolutionary relationships between the RPV isolates were inferred from the available unique F gene PCR products using maximum likelihood, as in Figure 6. The sequences used were the unique 322 base sequences obtained after combining the full length genomes published or available in the database(marked *), the full length genomes presented in this study, and the 322 base fragments from various diagnostic samples received in the laboratory over the years and previously used in describing the various RPV lineages (marked #). The figure shows the maximum likelihood tree in which the robustness of the resultant branches was assessed by the percentage of replicate bootstrapped trees (1000 replicates) in which that grouping of taxa occurred. The scale bar calibrates the evolutionary distance between isolates (branch lengths) in substitutions per site.

Table S2 Sequences of named primers used in these studies

Name	Sequence
**RACE1**	ATCGGRACAAYRATGACATG
**RACE2**	ATCRGGGTCATCRGTGAT
**RACE3**	TCCACCARYTTAATGCTGA
**RACE4a**	GCTYAAGCGTGARTGGCT
**RACE5**	GAAARTTYTGGGGTTACAT
**RACE6**	TGCTGAGYAGCAGGCAG
**Q1**	GAGGACTCGAGCTCAAGC
**QT**	CCAGTGAGCAGAGTGACGAGGACTCGAGCTCAAGCTTTTTTTT TTTTTTTTTVN
**M13RACE7c**	AAGTAAAACGACGGCCAGTYGATAGGATCTTGAATCCTAAGT
**M13FOR**	GTAAAACGACGGCCAG
**RPV.GC_F2**	AGTACWGGCAAAATGAGCAAGAC
**RPV.Frag3R**	TGRYCCTAAGTTTTGTTTRGTATT
**RPV.Frag4F**	CAYRAATACYAAACAAAACTTAGG
**RPV.R2**	CAGTGGATCTGGCCAGTG
